# Epileptogenesis following Kainic Acid-Induced Status Epilepticus in Cyclin D2 Knock-Out Mice with Diminished Adult Neurogenesis

**DOI:** 10.1371/journal.pone.0128285

**Published:** 2015-05-28

**Authors:** Ilona Kondratiuk, Gabriela Plucinska, Diana Miszczuk, Grazyna Wozniak, Kinga Szydlowska, Leszek Kaczmarek, Robert K. Filipkowski, Katarzyna Lukasiuk

**Affiliations:** 1 Laboratory of Neurobiology, Nencki Institute of Experimental Biology, Polish Academy of Sciences, Warsaw, Poland; 2 Laboratory of Epileptogenesis, Nencki Institute of Experimental Biology, Polish Academy of Sciences, Warsaw, Poland; 3 Laboratory of Biological Psychology, University of Finance and Management in Warsaw, Warsaw, Poland; 4 Behavior and Metabolism Research Laboratory, Mossakowski Medical Research Centre, Polish Academy of Sciences, Warsaw, Poland; University of Modena and Reggio Emilia, ITALY

## Abstract

The goal of this study was to determine whether a substantial decrease in adult neurogenesis influences epileptogenesis evoked by the intra-amygdala injection of kainic acid (KA). Cyclin D2 knockout (cD2 KO) mice, which lack adult neurogenesis almost entirely, were used as a model. First, we examined whether status epilepticus (SE) evoked by an intra-amygdala injection of KA induces cell proliferation in cD2 KO mice. On the day after SE, we injected BrdU into mice for 5 days and evaluated the number of DCX- and DCX/BrdU-immunopositive cells 3 days later. In cD2 KO control animals, only a small number of DCX+ cells was observed. The number of DCX+ and DCX/BrdU+ cells/mm of subgranular layer in cD2 KO mice increased significantly following SE (p<0.05). However, the number of newly born cells was very low and was significantly lower than in KA-treated wild type (wt) mice. To evaluate the impact of diminished neurogenesis on epileptogenesis and early epilepsy, we performed video-EEG monitoring of wt and cD2 KO mice for 16 days following SE. The number of animals with seizures did not differ between wt (11 out of 15) and cD2 KO (9 out of 12) mice. The median latency to the first spontaneous seizure was 4 days (range 2 – 10 days) in wt mice and 8 days (range 2 – 16 days) in cD2 KO mice and did not differ significantly between groups. Similarly, no differences were observed in median seizure frequency (wt: 1.23, range 0.1 – 3.4; cD2 KO: 0.57, range 0.1 – 2.0 seizures/day) or median seizure duration (wt: 51 s, range 23 – 103; cD2 KO: 51 s, range 23 – 103). Our results indicate that SE-induced epileptogenesis is not disrupted in mice with markedly reduced adult neurogenesis. However, we cannot exclude the contribution of reduced neurogenesis to the chronic epileptic state.

## Introduction

The generation of new neurons occurs in the dentate gyrus of the hippocampal formation throughout the lifespan of adult mammals [1,2]. Adult neurogenesis was proposed to play a role in epilepsy after increased neurogenesis in the dentate gyrus was observed in human patients as well as in experimental models of epilepsy, including status epilepticus (SE)-induced epilepsy [3–5]. In addition, SE induces the ectopic localization and morphological abnormality of newborn neurons, which may contribute to network hyperexcitability [4, 6–9]. Although disturbances in neurogenesis accompany epilepsy, the contribution of neurogenesis to epileptogenesis is not clear, especially because the integration of individual newborn neurons into the epileptic brain is complex and heterogeneous [10].

In this study, we aimed to determine if a substantial decrease in adult neurogenesis influences epileptogenesis evoked by intra-amygdala injection of kainic acid (KA). We used cyclin D2 knockout mice (cD2 KO mice) as a model because they almost entirely lack adult neurogenesis due to defects in progenitor proliferation [11–13].

## Methods

Cyclin D2 knockout mice (cD2 KO) [14] were obtained from the animal facility at the Nencki Institute. They were backcrossed for more than 10 generations into C57BL/6 and FVB backgrounds. Heterozygotic (cD2+/-) mice from these lines were used to generate C57BL/6 x FVB (50% x 50%) cD2 KO (-/-) and wild type (wt; +/+) littermates, as recommended for behavioral analysis of mice [15]. Animals at the age of 16–18 wks were used in all experiments. All animal procedures were approved by the Ethics Committee on Animal Research at the Nencki Institute and conducted in accordance with the guidelines set by the European Council Directive 86/609/ECC.

For evaluation of KA-induced early neurogenesis, SE was triggered in both male and female cD2 KO mice (n = 4) and their wt littermates (n = 5) by injection of KA (0.3 μl of 0.77 mg/ml at 10 nl/s) into the right basolateral amygdala (AP -2.0 mm, L -3.2 mm and DV -5.2 mm) under isoflurane anesthesia (1.5–2.0%). All animals enrolled in the experiment had generalized SE lasting 89.9±9.9 min, for wt mice, and 98±18.6 min, for cD2 KO mice, as determined by the presence of behavioral seizures scored at 4 to 6 according to modified Racine score for mice [16]. Corresponding controls (n = 4) received an injection of 0.9% NaCl using the same parameters as the KA-treated groups. Intra-amygdala injections were followed by intraperitoneal BrdU injections (50 mg/kg at 10 mg/ml) every 24 h over 5 days starting 24 h after KA or NaCl injection [17]. Three days after the last BrdU injection, mice were injected with a pentobarbital overdose (Morbital, Biowet; 150 mg/kg, i.p.) and perfused with 0.9% NaCl followed by 4% paraformaldehyde (PFA) in 0.1 M sodium phosphate buffer, pH 7.4. Brains were isolated and post-fixed in 4% PFA and were cryoprotected with 30% sucrose in 0.02 M potassium phosphate-buffered saline (KPBS) solution.

For immunohistochemical staining, brains were sectioned in the coronal plane (25 μm, 1-in-5 series) on a cryostat. Sections were stored in a cryoprotectant tissue-collecting solution (TCS; 30% ethylene glycol, 25% glycerol in 0.05 M sodium phosphate buffer) at -20°C. Double immunohistochemical staining for DCX (doublecortin) and BrdU (5-bromo-2-deoxyuridine) was performed according to standard protocol using primary antibodies against BrdU (rat monoclonal anti-BrdU, 3H578, sc-70441, Santa Cruz, 1:200) and DCX (goat polyclonal anti-DCX, C-18, sc-8066, Santa-Cruz, 1:200). Donkey anti-rat Alexa 488 (Invitrogen, A21208, 1:300) and donkey anti-goat Alexa 555 (Life technologies, A-21432, 1:1000) were used as secondary antibodies. Images were collected on a Leica Spinning Disc Confocal Microscope. Images were generated by Z stacking contiguous images using the maximal intensity projection function in ImageJ software. DCX positive (DCX+) and double-stained cells (DCX/BrdU+) in the ipsi- and contralateral subgranular layer of the dentate gyrus were counted manually from four sections per animal at 375 μm apart, starting approximately -1.46 from bregma. The length of the subgranular layer was determined with ImageJ software by manually drawing a line along the subgranular layer through the entire dentate gyrus on the maximal intensity projections. Images were analyzed in a blind manner with respect to the experimental group. Data are presented as number of DCX+ or DCX/BrdU+ cells/length of subgranular cell layer (mm). For evaluation of neurodegeneration, Nissl stained sections from the same animals were used.

For EEG (electroencephalography) recording, homemade electrodes consisting of stainless steel screws (#00-96X1/16, Plastic One Inc., Roanoke, VA), magnet wire and socket contacts (#E363/0, Plastic One Inc., Roanoke, VA) were implanted bilaterally in the skull over the frontal cortex as recording electrodes and over the cerebellum as reference and ground electrodes in mice anesthetized with isoflurane (1.5–2.0%). Male mice were used for this experiment. SE was evoked as described above. After surgery, animals were connected to an EEG recording system for 16 days of continuous EEG recording (Comet, Grass Technologies, West Warwick, RI). EEG recordings were analyzed manually (TWinn EEG, Grass Technologies, West Warwick, RI). Spontaneous seizures were identified from EEG recordings by browsing the EEG on the computer screen. An electrographic seizure was defined as a high-frequency (>8 Hz), high-amplitude (>2x baseline) discharge lasting for at least 5 s. Duration of the initial SE was available for 14 wt and 10 cD2 KO mice. Latency period to the first spontaneous seizures as well as frequency and duration of the spontaneous seizures were measured only for animals that did not lose head sockets until the 16th day post-KA injection or later (n = 15 of wt and n = 12 of cD2 KO mice). Nine wt and 8 cD2 KO mice that lost head sockets or died earlier were not included in this analysis.

Statistical analysis was performed using Sigma Stat 3.5 (Systat Software, Inc.). The minimal level of significance was p<0.05. The chi-square test was used to compare the number of animals with seizures within groups. SE duration was analyzed with the t-test. The Mann-Whitney test (for independent groups) was used for the data that violated the assumptions of the t-test.

## Results

**Fig 1:**
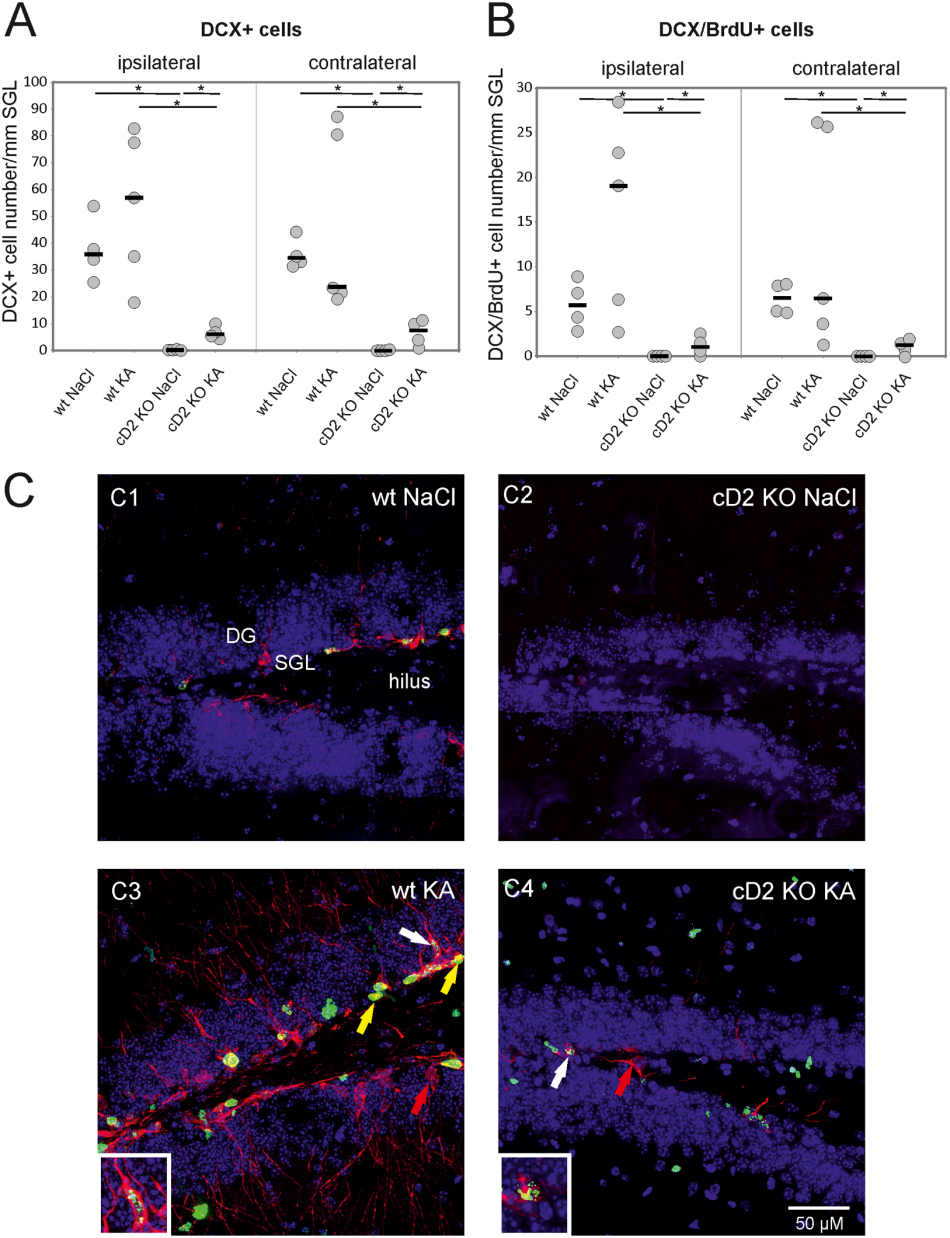
Kainic acid-induced early neurogenesis in the dentate gyrus of wt and cD2 KO mice. **(A)** Number of DCX+ cells/mm of subgranular layer of the ipsilateral and contralateral dentate gyrus. **(B)** Number of DCX/BrdU+ cells/mm of subgranular layer of the ipsilateral and the contralateral dentate gyrus. Each circle represents one animal and horizontal bars indicate median values. **(C)** Representative BrdU (green) and DCX (red) double immunostaining in wt and cD2 KO mice following NaCl (**C1, C2**, respectively) or kainic acid injection (**C3, C4**, respectively); * p<0.05 of Mann Whitney U test; yellow arrow—double stained, DCX/BrdU+ cells; red arrow—DCX+/BrdU- cells; white arrow and inserts in C3 and C4—DCX/BrdU+ cells with fragmented nucleus indicating apoptosis; cD2—cyclin D2; DCX—doublecortin; BrdU—5-bromo-2'-deoxyuridine; DG—dentate gyrus; SGL—subgranular layer; KO—knock-out; wt—wild type.

To determine if SE induced by intra-amygdala injection of KA leads to the induction of hippocampal cell proliferation, we evaluated the number of DCX+ and DCX/BrdU+ cells in the subgranular layer of the dentate gyrus 8 days after KA treatment (Fig 1). The median number of DCX+ cells/mm of subgranular cell layer in wt control mice was 35.8 (range 25.4–53.8) ipsilaterally and 34.5 (range 31.8–44.5) contralaterally. In KA-treated wt mice, the median number of DCX+ cells/mm of subgranular cell layer was 56.9 (range 17.8–82.7) ipsilaterally and 23.8 (range 19.6–87.6) contralaterally. There was no significant difference in the number of DCX+ cells between control and KA-treated wt mice (p>0.05, Mann-Whitney *U* test). In cD2 KO control animals, only occasional DCX+ cells were observed. The median number of DCX+ cells/mm of subgranular layer was 0.2 (range 0.0–0.4; p<0.05 compared to wt controls) ipsilaterally and 0.3 (range 0.1–0.7; p<0.05 compared to wt controls) contralaterally. KA treatment significantly increased (p<0.05 compared to cD2 KO controls) the number of DCX+ cells/mm of subgranular layer to 6.1 (range 4.3–10.0) ipsilaterally and 7.5 (range 1.8–11.9) contralaterally. This was still significantly fewer cells than in KA-treated wt mice (p<0.05).

**Fig 2:**
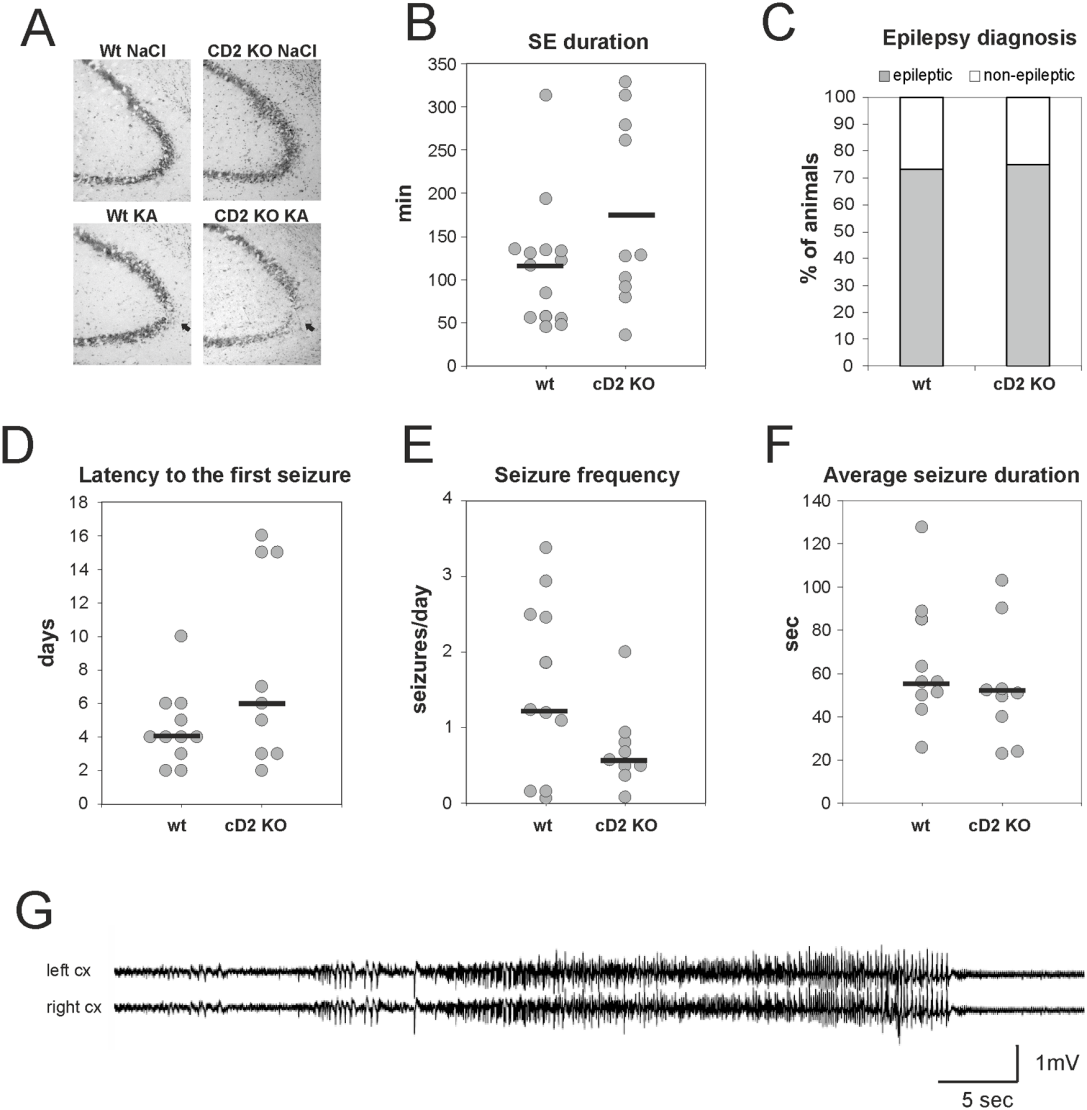
Epileptogenesis in wt and cD2 KO mice following intra-amygdala kainic acid injection. **(A)** Neurodegeneration in CA3 of the hippocampus at 8 d after KA-induced status epilepticus. **(B)** Duration of status epilepticus, **(C)** percent of animals developing epilepsy, **(D)** latency to the first spontaneous seizure, **(E)** seizure frequency in epileptic mice and **(F)** average spontaneous seizure duration in wt and cD2 KO mice following intra-amygdala kainic acid injection. **(G)** An example of an electrographic seizure detected in a cD2 KO animal. Arrows in A indicate the area of neuronal loss. Each circle in B and D-F represents one animal, and horizontal bars indicate mean (B) or median (D-F) values; cx—cortex, KO—knock-out, SE—status epilepticus, wt—wild type.

The median number of DCX/BrdU+ cells/mm of subgranular cell layer in wt control mice was 5.7 (range 2.8–8.9) ipsilaterally and 6.5 (range 5.0–8.2) contralaterally. In KA-treated wt mice, the median number of DCX/BrdU+ cells/mm of subgranular cell layer was 19.1 (range 2.7–28.4) ipsilaterally and 6.5 (range 1.4–26.2) contralaterally. As with the number of DCX+ cells there was no significant difference in the number of DCX/BrdU+ cells between control and KA-treated wt mice (p>0.05, Mann-Whitney *U* test). In cD2 KO control animals, no DCX/BrdU+ cells were found (p<0.05 compared to wt controls). A significant increase was observed following KA treatment (p<0.05 compared to cD2 KO controls). The number of DCX/BrdU+ cells/mm of subgranular layer was 1.0 (range 0.0–2.5) ipsilaterally and 1.2 (range 0.0–2.0) contralaterally. The number of DCX/BrdU+ cells was significantly lower than in KA-treated wt mice both ipsilaterally and contralaterally (p<0.05). Interestingly, in both wt and cD2 KO KA-treated mice, we observed DCX/BrdU+ cells with fragmented nuclei, indicating ongoing cell death (indicated by white arrows in Fig 1C3 and 1C4). Hippocampal damage observed at 8 d after KA injection in wt and cD2 KO mice was mild and localized to CA3 (Fig 2A), as previously described for this model by Tanaka et al.[18].

In mice used for EEG recordings, the severity of SE was evaluated by EEG. Following stereotactic injection of KA into the amygdala, mice entered SE as the effect of anesthesia receded. SE lasted 116±72 min in wt mice and 175±109 min in cD2 KO mice, and its duration did not differ between groups (p>0.05, t-test, Fig 2B).

The significant reduction in adult neurogenesis did not prevent the development of epilepsy following KA-induced SE in cD2 KO mice. Over the course of 16 days of monitoring, the number of animals in which epilepsy was diagnosed did not differ between wt and cD2 KO mice (Fig 2C). Seizures were observed in 9 out of 12 cD2 KO mice and in 11 out of 15 wt mice (p>0.05, chi-square test). In addition, latency to the first spontaneous seizure was not affected (Fig 2D). The median latency to the first spontaneous seizure in cD2 KO mice was 6 days (range 2–16) and in wt mice, it was 4 days (range 2–10) following KA-induced SE (p>0.05, Mann-Whitney *U* test). The difference in seizure frequency between cD2 KO and wt mice with diagnosed epilepsy was not statistically significant. Median seizure frequency/day in cD2 KO mice was 0.57 (range 0.1–2.0) and in wt mice was 1.23 (range 0.1–3.4) (p>0.05, Mann-Whitney *U* test, Fig 2E). There was also no difference between groups in average seizure duration during the 16-day monitoring period. Median of average seizure duration in cD2 KO animals was 51 s (range 23–103), while in wt mice it was 56 s (range 25–127; p>0.05, Mann-Whitney *U* test, Fig 2F).

## Discussion

In this study, we show no significant difference between the development of epilepsy and seizure number and frequency during early epilepsy in control mice and cD2 KO mice with greatly reduced adult neurogenesis. Therefore, adult neurogenesis appears not to be obligatory for SE-induced epileptogenesis.

Neurogenesis increases in the adult rodent hippocampus following brain damage, also after epileptogenic brain insults and seizures [19, 20]. Neurons born in response to such stimuli are capable of integrating into existing neuronal networks but have been shown to display abnormalities, including misguided localization, improper dendrite and synapse formation, and altered synaptic excitability [5, 19, 20]. These newborn neurons could potentially be an anatomical substrate for epileptogenesis, but their role in this process remains elusive (recently reviewed in [19]).

In our study, we used cD2 KO mice, which show remarkably (95%) reduced adult hippocampal neurogenesis [11–13, 21–23]. These mice have been used previously as a tool for investigating the role of newly born neurons (discussed in [24]). The results obtained with cD2 KO mice suggest that adult brain neurogenesis is not relevant to the efficacy of the antidepressant fluoxetine [23] as well as for learning and memory in general [13], while the role of adult neurogenesis in particular aspects of learning [25, 26], smell detection [13], species-typical behavior [22], and alcohol consumption [27] have been suggested or confirmed.

We showed previously that reduced neurogenesis in cD2 KO mice is limited to the central nervous system, and the number of new cells was not increased by either an enriched environment [11] or by fluoxetine treatment of mutant mice [23]. Here, we show for the first time that limited induction of early neurogenesis is possible in cD2 KO mice. Contrary to expectation, we did not detect a statistically significant increase in the number of DCX+ or DCX/BrdU+ cells in KA-treated wt mice. This may be due to the high variability in the number of newborn cells in this group of animals. It is possible that the low number of newborn cells in wt animals results from increased cell death caused by an unfavorable local microenvironment [28–30]. This is supported by the frequent presence of apoptotic DCX+ and DCX/BrdU+ cells in those animals. Importantly, we did detect a statistically significant increase in the number of DCX+ and DCX/BrdU+ cells in cD2 KO animals following KA-induced SE. It should be noted, however, that even a stimulus as strong as SE leads to the generation of only occasional newborn cells in cD2 KO animals, and their number does not reach that observed in untreated wt animals.

Previous studies aiming to prevent epileptogenesis by suppressing neurogenesis after epileptogenic insult have produced conflicting results. An elongation of the latency period between pilocarpine-induced SE and the first spontaneous seizure was observed following treatment with cytosine-beta-D-arabinofuranoside or celecoxib, a cyclooxygenase-2 inhibitor [31, 32]. However, these compounds also affected neurodegeneration and gliosis, which could be responsible for the attenuation of epileptogenesis. γ -irradiation, a more selective method of suppressing excessive neurogenesis, was used in the kindling model of epileptogenesis and lead to a paradoxical enhancement in the excitability of hippocampal neurons and slightly accelerated hippocampal kindling [33]. On the other hand, Pekcec et al. [34] found no effect of targeted irradiation of the hippocampus on the progression of amygdala kindling, suggesting that ablation of neurogenesis does not affect epileptogenesis.

Interestingly, the neurons most affected are those born shortly before or just after the damaging stimulus, and they require a few weeks to fully mature and integrate into the hippocampal network [5, 9]. Thus, it is less likely that the integration of those neurons is responsible for epileptogenesis in our model, as the first spontaneous seizures in wt animals are observed much earlier. Additionally, cD2 KO mice, which lack neurons born before KA treatment and display proliferation of progenitors at barely detectable levels after KA treatment, do develop epilepsy.

One limitation of this study is that cD2 KO mice are not yet fully characterized in terms of aberrant network excitability or other pathologies that could influence epilepsy development. For example, the reduction of parvalbumin interneurons and the reduced frequency of GABA-mediated inhibitory postsynaptic currents that have been detected in the cortex of cD2 KO mice could influence epileptogenesis [35]. Interestingly, parvalbumin interneurons undergo neurodegeneration in SE models of epilepsy [36, 37]. It is difficult to speculate whether the defective cortical inhibition observed in cD2 KO animals might influence the impact of diminished neurogenesis on epileptogenesis progression.

Our study using cD2 KO mice, which display markedly limited adult brain neurogenesis and in which proliferation of neural progenitors in the subgranular layer is negligible after KA-induced SE, indicates that neurogenesis is not required for SE-induced epileptogenesis and early epilepsy. However, based on our study, we cannot exclude a possible contribution of adult neurogenesis to the chronic epileptic state.
